# Characterization of Breast Microcalcifications Using Dual-Energy CBCT: Impact of Detector Configuration on Imaging Performance—A Simulation Study

**DOI:** 10.3390/s25226853

**Published:** 2025-11-09

**Authors:** Evangelia Karali, Christos Michail, George Fountos, Nektarios Kalyvas, Ioannis Valais

**Affiliations:** Radiation Physics, Materials Technology and Biomedical Imaging Laboratory, Department of Biomedical Engineering, University of West Attica, Ag. Spyridonos, 12210 Athens, Greece; ekarali@uniwa.gr (E.K.); cmichail@uniwa.gr (C.M.); gfoun@uniwa.gr (G.F.); nkalyvas@uniwa.gr (N.K.)

**Keywords:** CBCT, dual-energy CBCT, microcalcifications, CNR, HAp, photon-counting detectors, GAGG, CZT, breast imaging

## Abstract

**Highlights:**

**What were the main findings?**

- CZT and GAGG crystals exhibited higher CNR values than CsI.
- HAp’s CNR values were high, as expected.

**What are the implications of the main findings?**

- CZT and GAGG crystals could provide an excellent alternative to CsI.
- HAp’s CNR values enable it to be distinguished from other types of microcalcifications.

**Abstract:**

Microcalcifications (HAp, CaCO_3_, and CaC_2_O_4_) in breast tissue may indicate malignancy. Early-stage breast cancer diagnosis may benefit from the clinical application of dual-energy techniques. Dual-energy cone-beam computed tomography (CBCT) could strongly contribute to an accurate diagnosis, especially in dense breasts. This study focused on photon-counting detector alternatives to the standard cesium iodide (CsI) that CBCT currently relies on and investigated potential advantages over the employed CsI scintillators. Denser detector materials with a higher effective atomic number than CsI could improve image quality. A micro-CBCT was simulated in GATE using seven different detector configurations (CsI, bismuth germanate (BGO), lutetium oxyorthosilicate (LSO), lutetium–yttrium oxyorthosilicate (LYSO), gadolinium aluminum gallium garnet (GAGG), lanthanum bromide (LaBr_3_), and cadmium zinc telluride (CZT)) and four breast tissue phantoms containing microcalcifications of both type I and type II. The dual-energy methodology was applied to planar and tomographic acquisition data. Tomographic data were reconstructed using filtered backprojection (FBP) and the ordered-subsets expectation-maximization (OSEM) algorithm. Image quality was measured using contrast-to-noise ratio (CNR) values. Both monoenergetic and polyenergetic models were considered. CZT and GAGG crystals presented higher CNR values than CsI. HAp microcalcifications exhibited the highest CNR values, which, when accompanied by OSEM, could be distinguished for classification. Detector configurations based on CZT or GAGG crystals could be adequate alternatives to CsI in dual-energy CBCT.

## 1. Introduction

The estimated incidence and mortality rates of breast cancer in women vary across EU-27 countries, reaching up to 190 new cases and 45 deaths per 100,000 women [1]. The factors contributing to these observed geographical variations in breast cancer incidence and mortality include the implementation of organized breast cancer-screening programs and differences in the prevalence and distribution of major risk factors.

Microcalcifications in the breast are deposits of calcium oxalate (Ca_2_C_2_O_4_), calcium phosphate (Ca_2_(PO_4_)_2_) in the form of hydroxyapatite (HAp), and calcium carbonate (CaCO_3_), with diameters smaller than 0.5 mm. Their presence in imaging is now considered indicative of pre-cancerous or cancerous lesions [2,3,4,5,6]. Accurate depiction and quantification of microcalcifications is vital for a reliable medical diagnosis [7,8,9].

In mammography, microcalcifications appear as tight clusters of tiny white dots. Their shape often indicates the patient’s malignancy risk. “Popcorn” or “eggshell” shapes usually suggest low risk, whereas irregular or linear patterns are associated with malignancy [10]. Human breast composition and density vary globally. In mammography, fatty tissue appears dark, while dense tissue and tumors appear white. Thus, in dense breasts, both microcalcifications and tumors may go undetected due to a lack of contrast with surrounding tissue. Dense breast tissue also poses a higher cancer risk [11,12,13].

Therefore, mammography has low sensitivity and accuracy in dense breasts and often leads to false diagnoses. Three-dimensional tomosynthesis has similar limitations.

Cone-beam computed tomography (CBCT) with X-rays is at the forefront of modern research aiming to design small-scale CT systems suitable for breast imaging. Current research shows that the radiation dose can be comparable to traditional mammography, but without breast compression. CBCT also appears to offer better resolution in dense breasts [14].

Thus, developing a CBCT system with sufficient resolution and high sensitivity for dense breast imaging is of strong scientific interest.

CBCT technology relies on detectors that use a scintillator (e.g., CsI:Tl doped with thallium) to convert X-ray energy into light, followed by conversion to electrical signals. CsI:Tl has a high light output (>60,000 ph/MeV at room temperature) [15] and emits light at around 560 nm, matching the spectral sensitivity of photodiodes [16]. However, CsI:Tl has a relatively slow response time (0.6–0.9 µs), which may be inadequate for high-count-rate X-ray imaging [16]. Moreover, it remains hygroscopic even when doped with Tl, limiting its use in high-humidity environments [17,18]. CsI:Tl also exhibits significant afterglow, which introduces image blur in fast imaging applications [19].

Image quality depends on the signal-to-noise ratio (SNR) or contrast-to-noise ratio (CNR). The SNR (or CNR) can be improved by increasing photon counts (e.g., a higher mAs, but at the cost of a higher dose) or by increasing the detective quantum efficiency (DQE) [20], without increasing the dose [21]. The DQE can also be enhanced using thicker detectors or materials with higher X-ray attenuation coefficients (μ), which are influenced by atomic number and material density [22,23,24].

Hence, comparing scintillators and semiconductors with higher densities than CsI (e.g., bismuth germanate (BGO), lutetium oxyorthosilicate (LSO), lutetium–yttrium oxyorthosilicate (LYSO), gadolinium aluminum gallium garnet (GAGG), and cadmium zinc telluride (CZT)) is of scientific interest for clinical CBCT.

Simultaneously, international research has shown that imaging the same region under two different X-ray energy spectra enhances structure visibility and the CNR. This dual-energy CT (DECT) technique exploits the energy dependence of tissue attenuation [25]. Lower energy ranges tend to enhance contrast between tissues. Thus, DECT imaging significantly improves the visualization of microcalcifications [26,27,28,29,30,31,32,33]. Dual-energy imaging can be implemented with photon-counting detectors (PCDs) that can differentiate energy windows within a single scan using energy thresholds, improving the CNR and spatial resolution and enabling dose reduction [34,35]. Consequently, photon-counting detectors may enable DECT in clinical environments, where there are concerns about patient dose, and it is typically not used. DECT is highly significant in diagnostic radiology, particularly for breast imaging [36,37,38].

This study presents a comparative evaluation of various modern detector materials in a micro-CBCT system combined with DECT imaging. The imaging performance of each detector configuration was assessed to identify the optimal combination. Both scintillators and semiconductors were evaluated as potential X-ray energy converters. The scintillators used were BGO, LSO:Ce and LYSO:Ce (both doped with Ce), CsI:Tl (doped with Tl), GAGG:Ce, and lanthanum bromide (LaBr_3_:Ce). Additionally, the semiconductor CZT was evaluated. Clinical and experimental X-ray systems often use LYSO:Ce [39], while LSO:Ce and BGO are found in clinical and experimental gamma-ray systems [21]. These materials are dense (about 7 g/cm^3^) with a high light output (BGO: 30%; LSO:Ce: 85%; and LYSO:Ce: 85% of that of NaI:Tl) [40,41]. GAGG is attracting interest due to its high density (6.63 g/cm^3^ [40]), good light yield, and fast X-ray response (50–60 ns). It emits at 540 nm, which couples well with avalanche photodiode arrays [41]. Although LaBr_3_:Ce is hygroscopic, it is notable for its fast response (~20 ns [39,40,41,42,43]). Similarly, CZT is under investigation for CT systems due to its low electronic noise (nA range) and high SNR [44]. All these crystals’ properties are presented in Table 1.

The high density and effective atomic number Z_eff_ of these materials (higher than those of CsI:Tl) improve DQE and can yield better image quality without increasing dose. New CBCT detector configurations that increase the SNR while maintaining a low patient dose are of great scientific interest [45]. Therefore, this study aimed to investigate promising scintillator materials that can improve CNRs compared with standard CsI:Tl detectors when used with DECT methodology.

**Table 1:** Crystals’ properties [46,47].

Material	Effective Z (Z_eff_)	Density (g/cm^3^)	Light Yield (Photons/MeV)	Typical Energy Resolution @ 662 keV (FWHM)
CsI (Tl or Na, or undoped)	~54 (for CsI)	~4.51 (CsI)	~ 54,000 ph/MeV (for CsI:Tl under ideal coupling) (varies)	~6–8% (in good coupling)
BGO (Bi_4_Ge_3_O_12_)	~75 (weighted by Bi, Ge, and O)	7.13	~ 8000–10,000 (relatively low)	~10–12% (or worse)
LSO (Lu_2_SiO_5_:Ce)	~65	~7.4	~ 25,000–32,000	~9–10%
LYSO (Lu_2_(1–x)Y_2_xSiO_5_:Ce)	~63–65 (depending on Y fraction)	~7.1	~ 25,000–32,000 (similar to LSO)	~8–10%
LaBr_3_:Ce	~ 47 (La + Br)	5.08	~ 63,000 ph/MeV (or ~ 63 ph/keV)	~2.6%
GAGG:Ce (Gd_3_Al_2_Ga_3_O_12_:Ce)	~ 54.4 (often quoted)	~6.63 g/cm^3^	~ 46,000 ph/MeV	~4.9%
CZT (CdZnTe; semiconductor detector)	Not a scintillator, but has a high effective Z (Cd, Zn, and Te)	5.76 g/cm^3^	Not scintillation	~ 0.5–2%

## 2. Materials and Methods

### 2.1. Micro-CT System Simulation

**Figure 1:**
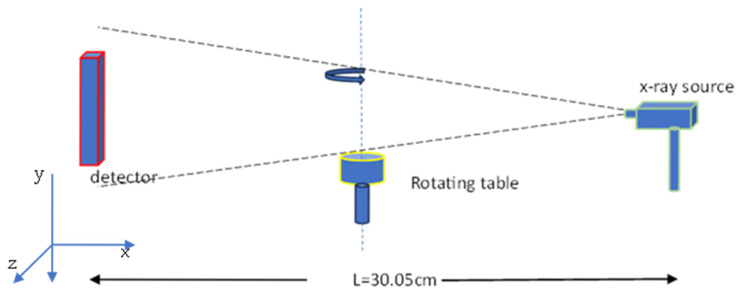
Schematic diagram of a cone-beam micro-CT X-ray system.

**Figure 2:**
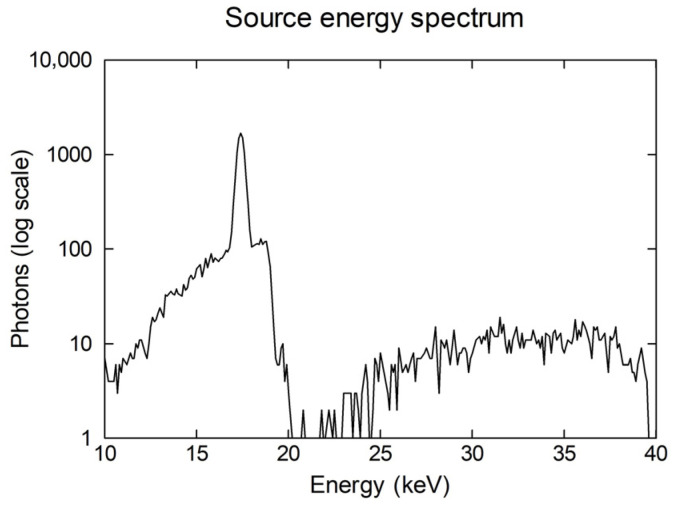
Energy spectrum of X-ray source.

Figure 1 presents a schematic diagram of the cone-beam micro-CT system. It consisted of a micro-focus X-ray source with an emission angle of 6.8°. The energy spectrum of the source ranged from 10 to 40 keV and is shown in Figure 2. The activity of the X-ray source was set to 0.35 MBq or 9.46 μCi. GATE v9.2.1 is built for nuclear medicine medical imaging system simulation and uses source radioactivity even for X-ray medical systems [48]. GATE is a simulation tool based on GEANT4 11.3.2, widely used in high-energy physics. The object under examination was placed on a rotating table (360° around the vertical axis), 15 cm away from the source. The table rotation was set at 1°/s from 0° to 360°. The table rotated around the y-axis; however, the y-values increased toward the bottom of the table. The detector consisted either of a semiconductor or a scintillator segmented into 100 × 100 pixels, with overall dimensions of 50 × 50 × 1 mm^3^. The pixel size was 0.5 × 0.5 × 1 mm^3^.

The micro-CBCT detection system was simulated using six different scintillators: BGO, LSO:Ce, LYSO:Ce, CsI:Tl, GAGG:Ce, and LaBr_3_:Ce. Additionally, CZT semiconductor detectors were also studied. Generally, materials with short decay times and high light output are preferred in medical imaging applications. Each detector was coupled to the same electronic signal processing system. A simple pulse analysis scenario with an energy threshold of 10 keV was chosen for the simulation.

The acronym stands for the GEANT4 application for tomographic emission. It is an advanced Monte Carlo simulation software capable of modeling radiation interactions (such as attenuation, scattering, and photon absorption), detector configurations, acquisition protocols, and signal processing during nuclear medicine imaging examinations. This makes GATE a powerful tool for designing new imaging systems, optimizing examination protocols, and investigating novel detection and signal-processing methodologies. The latest version of GATE also includes simulation routines specifically developed for X-ray computed tomography applications [48].

The electronic processing module consisted of an adder to sum all the hits that occurred within the same crystal, followed by a readout module with the readout depth set to 2. This readout depth value ensured that the energy of a single event was the sum of the energies of the pulses inside each detector element. A cut-off threshold was also applied for energies below 10 keV. Data were stored in txt format. Three hundred and sixty text scripts were stored. Each script contained the number of collected photons for every detector pixel at one angle of rotation.

The size of the mammographic images was 256 × 256 pixels, while the tomographic image dimensions were 128 × 128 pixels.

For each acquisition, 100 sinograms were generated. Each sinogram corresponded to a specific detector pixel row. Thus, each 2D sinogram from the 3D dataset contained the number of detected photons for each tomographic ray and each rotation angle.

### 2.2. Evaluation Phantoms

#### 2.2.1. Type I: Ca_2_CO_4_ and CaCO_3_ Microcalcifications

**Figure 3:**
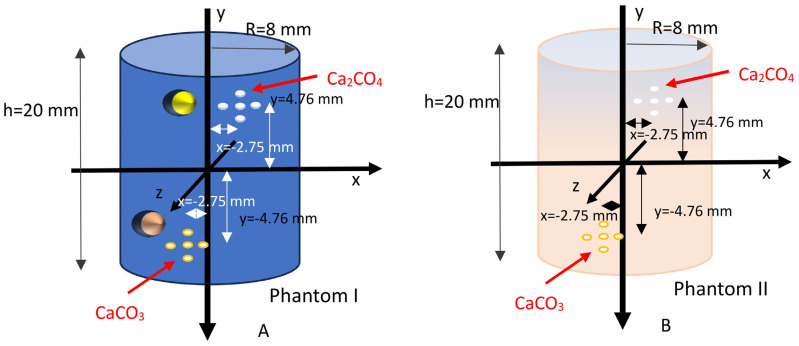
Breast phantoms: (**A**) breast phantom (phantom I) using water as a breast tissue simulator, with PVC and cortical bone spheres to increase the overall density; (**Β**) breast phantom (phantom II) using actual breast tissue. The Ca_2_CO_4_ and CaCO_3_ microcalcifications were placed in the same positions in both phantoms.

Two phantoms of similar geometry were used to evaluate the performance of the different detector configurations. The first (phantom I) was a breast phantom consisting of water and microcalcifications made of Ca_2_CO_4_ arranged into clusters on the upper part of the phantom and microcalcifications made of CaCO_3_ with an identical morphology on the lower part. There were PVC spheres and cortical bone in proximity to both types of microcalcification clusters, respectively. These materials had densities similar to those of the microcalcifications to simulate breast tissue samples classified as BI-RADS B or higher. The second phantom (phantom II) consisted of a common type of breast tissue sample that also contained microcalcifications, as in the first phantom. These two breast phantoms were used to investigate the CNR in a highly scattering environment (water or breast tissue) for each detector technology. The geometries of the two phantoms are shown in Figure 3.

#### 2.2.2. Type II: Hydroxyapatite (HAp) Microcalcifications

**Figure 4:**
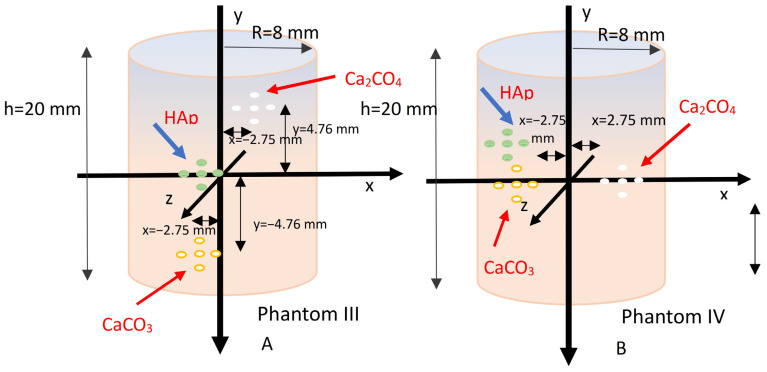
(**A**) Breast phantom III, with the three types of microcalcifications distributed at three different depths within the breast tissue (left). (**B**) Breast phantom IV, with the three types of microcalcifications placed at the same depth, centrally within the breast tissue.

Calcium hydroxyapatite (Ca_10_(PO_4_)_6_(OH)_2_), or HAp, belongs to type II microcalcifications. It has a molecular weight of 1004.6 g/mol and a density of 3.18 g/cm^3^. It contains phosphorus in addition to calcium, and it exhibits high X-ray attenuation coefficients for photon energies in the range of 25–40 keV [8,9,49]. Due to its significance in breast imaging, two breast phantoms (phantom III and phantom IV) were simulated, containing HAp, CaCO_3_, and CaC_2_O_4_. The phantoms differed in geometry and are shown in Figure 4.

### 2.3. Dual-Energy X-Ray Imaging Methodology

The dual-energy X-ray imaging method was implemented using the technique of two successive acquisitions at different X-ray tube voltages: 25 kV and 40 kV [50]. A rhodium (Rh) filter with a thickness of 50 μm was used for the low-energy acquisition at 25 kV. An aluminum (Al) filter with a thickness of 1 mm was applied for the high-energy acquisition at 40 kV. These filters were selected to absorb low-energy X-rays and shape the resulting spectra to be quasi-monoenergetic, ensuring that the emitted X-rays were primarily centered around spectral energies of either 25 keV or 40 keV.

In addition to the two main acquisitions with the phantoms at both energy levels, two corresponding reference acquisitions were simulated: one blank scan and another with the phantom, but without microcalcifications. The detected data for each case was obtained. By applying appropriate subtraction between these image sets, the resulting image (denoted as ${de}_{im}$) enhanced only the presence of microcalcifications, according to the following expression:

(1) $$\begin{array}{cc} {de}_{im} & =\left\{\log\left(\frac{N_{40\;keV}}{N_{0\_40\;keV}}\right)-\left(\frac{\mu_{40\;keV}}{\mu_{25\;keV}}\right)\times \left(\frac{Q_{40\;keV}}{Q_{25\;keV}}\right)\log\left(\frac{N_{25\;keV}}{N_{0\_25\;keV}}\right)\right\}-\{\log\left(\frac{N_{no\;microcalcification,40\;keV}}{N_{0_{40\;keV}}}\right)\\ & -\left(\frac{\mu_{40\;keV}}{\mu_{25\;keV}}\right)\times \left(\frac{Q_{40\;keV}}{Q_{25\;keV}}\right)\log\left(\frac{N_{no\;microcalcification,25\;keV}}{N_{0\_25\;keV}}\right)\} \end{array}$$

where $\log\left(\frac{N_{25\;keV}}{N_{0\_25\;keV}}\right)$ is defined for the image acquired with the phantom at the 25 keV energy window, normalized by the blank scan $N_{0\_25\;keV}$, and $N_{no\;microcalcification\_25\;keV}$ is the exponential image with the phantom but without microcalcifications. $N_{25\;keV}$ is the exponential image with the phantom containing microcalcifications, while the definitions for the 40 keV window are $N_{0\_40\;keV}$, $N_{no\;microcalcification,\_40\;keV}$, and $N_{40\;keV}$, respectively. Furthermore, $\frac{\mu_{40\;keV}}{\mu_{25\;keV}}$ is the ratio of the microcalcifications’ linear attenuation coefficients at 40 keV and 25 keV, respectively, and $\left(\frac{Q_{40\;keV}}{Q_{25\;keV}}\right)$ is the ratio of the X-ray photon absorption efficiency of the detector at 40 keV and 25 keV, respectively [51].

Equation (1) can also be applied at the sinogram level, meaning it is applied to the set of collected photons at each rotation angle and for all tomographic rays.

The linear attenuation coefficients of Ca_2_CO_4_ and CaCO_3_ were obtained from the corresponding tables provided by the National Institute of Standards and Technology (NIST) [52]. The photon absorption efficiencies of each detector crystal were calculated using Equation (2) [53]:

(2) $$Q_{E_{i}}=1-e^{-\mu_{E_{\iota }}x}$$

where *E_i_* is 25 or 40 keV, $\mu_{E_{\iota }}$ is the linear attenuation coefficient of the scintillator or semiconductor (in units of 1/cm) at energy *E_i_*, and *x* = 1 mm is the constant thickness of the scintillator or semiconductor.

The corresponding attenuation coefficients $\mu_{E_{\iota }}$ were also obtained from NIST [52].

In this study, a monoenergetic X-ray beam model was adopted. In the case of polyenergetic beams, the effective attenuation coefficients are calculated using Equation (3):

(3) $$\mu_{E_{\iota }}=\frac{\sum_{Emin}^{Emax}\mu_{E_{k}}I_{o_{k}}}{\sum_{Emin}^{Emax}I_{o_{k}}}$$

where $\mu_{E_{\iota }}$ is the effective linear attenuation coefficient of the scintillator or semiconductor at 25 or 40 keV; $I_{o_{k}}\;$ is the initial photon flux incident on the microcalcifications, which depends on the energy spectrum of the X-ray source; and *E_max_* and *E_min_* are the maximum and minimum photon energies in the X-ray spectrum, respectively [50,53].

### 2.4. Image Reconstruction

CBCT data, usually in clinical systems, are reconstructed using the Feldkamp–Davis–Kress (FDK) algorithm [54]. FDK reconstructs the whole 3D dataset, while FBP [45] assumes a 2D fan-beam geometry and reconstructs 2D slices sequentially, taking into consideration the 3D dataset. However, FDK also assumes that the geometry locally, around every voxel that is reconstructed, is a 2D fan-beam geometry; thus, the differences are almost negligible. Moreover, the source aperture is small, about 6.8°, which allows FBP to quickly produce an acceptable reconstructed image, without significant error or artifacts [55]. That was the reason why we chose FBP and reconstructed 2D tomograms slice by slice, with their further merging into a 3D image.

FBP is based on the Fourier theorem of the central section theorem, which connects projection data with image data via the Fourier transformation. The 1D Fourier transformation of a projection data in one angle of rotation φ is equal to the 2D Fourier transform of the image data along a projection line that forms an angle φ with the horizontal image axis (Equations (4) and (5)).

(4) $$f\left(x,y\right)=\frac{1}{N}\sum_{n=1}^{N}s^{\prime }\left(r,\varphi_{n}\right)$$

(5) $$\mathrm{where}\;s^{\prime }\left(r,\varphi_{n}\right)=\frac{1}{2\pi }{FT}^{-1}(FT\left(s\left(r,\varphi_{n}\right)\right)\times H\left(v\right))$$

${FT}^{-1}$ denotes the inverse Fourier transform; FT stands for the Fourier transform; $s\left(r,\varphi_{n}\right)$ is the projection data at angle $\varphi_{n}$; and $H\left(v\right)$ is a filter applied in the frequency v domain; x,y are cartesian coordinates; and r is the radial distance; finally, × denotes multiplication in the frequency domain.

FBP was implemented by applying a Hamming window to avoid blurring artifacts in the reconstructed images. Furthermore, high-frequency image noise was reduced, and the signal-to-noise ratio (SNR) was improved. FBP was applied in combination with bilinear interpolation. FBP is a widely used analytical reconstruction algorithm in tomographic imaging that offers fast computation and straightforward implementation. It applies a mathematical filter to projection data before backprojecting them into image space.

Additionally, the iterative OSEM (ordered subsets expectation maximization) algorithm [54] was also used for tomographic image reconstruction. The OSEM algorithm is an iterative statistical method that improves image quality by modeling the physics of data acquisition. OSEM divides projection data into subsets and iteratively refines the image estimate, resulting in better noise suppression, contrast recovery, and resolution, especially in low-count or limited-angle datasets [56]. Although it is computationally more intensive than FBP, OSEM is favored in clinical and research applications where quantitative accuracy and high image quality are essential [57].

Iterative methods usually rely on a linear relation between collected data *y* and image *x*, according to Equation (6):

(6) y=Ax

*A* is the system or probability matrix, which models all the physical phenomena during the data acquisition process (i.e., the X-ray scatter and attenuation) as well as the scanner’s geometric characteristics (the angle of rotation, object-to-detector distance, distance from the object to the X-ray source, detector’s pixel number, detector pixel size, image size, image pixel size, and angle of X-ray emission). Element $a_{ij}$ of matrix *A* represents the probability of an X-ray passing from image pixel *i* being detected by detector pixels *j* that define the LOR *j* (LOR (line of response)). Since *A* is not quadratic, *A*^−1^ cannot be calculated, and Equation (1) cannot be directly solved. Iterative techniques can reach an optimum image representation of the object under study to produce the optimum solution of Equation (1) via multiple iterations based on specific objective functions, relative to the physical phenomena of data acquisition [58].

The implementation of OSEM assumes that collected data follow Poisson statistics, with mean value $\sum_{i=1}^{N}a_{ij}x_{i}$, where N is the total image pixel number. There is no gold standard on the choice of subsets; for example, they can be sequentially ordered with no overlap between them, or they can be overlapping subsets with increasing size. In the kth iteration and for subset n, the iterative step for OSEM can be written as follows (Equation (7)):

(7) $$x_{i}^{k}=x_{i}^{k-1}\frac{1}{\sum_{j\in Sn}a_{ij}}\sum_{j\in Sn}\frac{a_{ij}y_{j}}{\sum_{i=1}^{N}a_{ij}x_{i}^{k-1}}$$

The system matrix was implemented according to an analytical formula, as described in [59]. The iterative OSEM (ordered subsets expectation maximization) algorithm was used for tomographic image reconstruction, with 24 subsets and 2 iterations applied to the 40 keV tomographic data, while the algorithm was implemented with 24 subsets and 1 iteration in the dual-energy application.

### 2.5. Image Quality

Image quality was assessed by calculating the CNR locally in the vicinity of the microcalcification area. ROIs of 3 × 3 pixels in size were used for both the microcalcifications and the background pixels. The CNRs were calculated according to Equation (8):

(8) $${CNR}_{mc\_object}=\frac{M_{mc\_object}-M_{background}}{\sigma_{background}}$$

where $M_{mc\_object}$ stands for the main microcalcification’s pixel intensity inside the selected ROI, and the main background intensity is $M_{background}$, and $\sigma_{background}$ stands for the standard deviation of the background intensity in the selected background ROIs [58]. Four CNR values were calculated for each type of microcalcification. The mean value from these four values was extracted and is presented in the Results Section.

Normalized root mean square error (NRMSE) [60] was also used to assess image quality. CsI-reconstructed data were considered as ground truth.

### 2.6. Image Segmentation Algorithm

The authors of [61,62] present a usual and well-established segmentation methodology to cluster microcalcifications according to the CNR values. It is applied to cases where data are limited and deep learning methods cannot be used. It is widely used on clinical data and especially in microcalcification detection [63].

### 2.7. Clustering

Agglomerative hierarchical clustering was chosen to group HAp microcalcifications. This method clusters detected features, such as centroids of detected objects, according to their spatial proximity. This unsupervised clustering method is suitable for use on clinical data for various features’ detection across slices or patients [64,65,66].

## 3. Results

Data were simulated for each X-ray energy using the aforementioned phantoms with and without microcalcifications. “Βlank” acquisitions were also performed at each energy without the phantoms in the camera’s field of view.

### 3.1. Mammography Planar Acquisitions

#### 3.1.1. Type I

**Figure 5:**
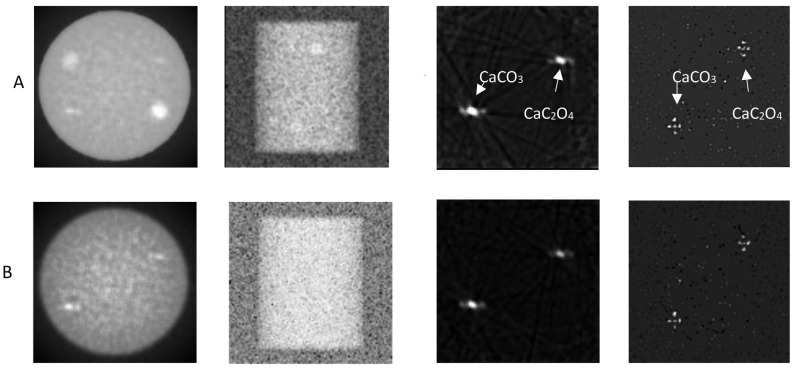
Mammographic images with CsI: left column a—mammography; column b—“planar mode” acquisition. Columns c, d—mammographic images with CsI using the “dual-energy” method: column c—mammographic view of the phantom; right column d—“planar mode” acquisition. Row (**A**) Breast phantom with water-equivalent simulator (phantom I) (Figure 3, left); row (**B**) breast phantom with standard breast tissue simulator (phantom II) (Figure 3, right).

Figure 5 (columns a and b) shows the mammographic images and the planar images at 45° of the simulated data derived from the phantoms in Figure 3. The X-ray maximum energy was set to 40 keV. These images were detected with a detector configuration based on CsI, which represents the clinical CBCT systems. The microcalcifications were barely distinguishable, and CNR values could not be extracted for either phantom.

Figure 5 (columns c and d) shows the application of the dual-energy technique at the image level for the mammographic images presented in Figure 5 (columns a and b). The monoenergetic beam model was used, with $\frac{\mu_{40\;keV}}{\mu_{25\;keV}}=0.29$, while the absorption ratios $\left(\frac{Q_{40\;keV}}{Q_{25\;keV}}\right)$ were calculated using Equation (2). In this case, the microcalcifications were clearly visible, and their morphology was distinguishable in the oblique-view images. The mean CNRs for the microcalcifications in the oblique-view (planar) images are presented in Table 2 for both phantoms Ι and ΙΙ. No CNR values over the Rose criterion [58] could be extracted from the transverse mammographic images. The Rose criterion assumes a CNR value of 3 to distinguish the object.

#### 3.1.2. Types I and II

**Figure 6:**
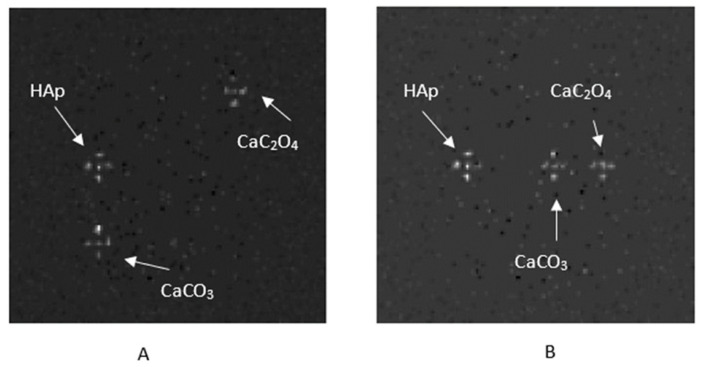
Application of the “dual-energy” method in planar mode for (**A**) phantom III (Figure 4, left) and (**B**) phantom IV (Figure 4, right) using a detector configuration based on a CZT crystal.

Figure 6 shows the application of the dual-energy technique at the image level for the breast phantoms in Figure 4 using a detector configuration based on a CZT crystal. The monoenergetic beam model was used, with $\frac{\mu_{40\;keV}}{\mu_{25\;keV}}=0.29$, while the absorption ratios $\left(\frac{Q_{40\;keV}}{Q_{25\;keV}}\right)$ were calculated using Equation (2). In this case, the microcalcifications (all three types) were clearly visible, and their morphology was distinguishable in the oblique-view images. Table 2 presents the mean CNRs for the microcalcifications in the oblique-view images for breast phantoms I and II, while Table 3 presents the mean CNRs for phantoms III and IV for FBP and OSEM.

### 3.2. Tomographic Data

The monoenergetic beam model was used. The dual-energy technique was applied directly to the tomographic data, with $\frac{\mu_{40\;keV}}{\mu_{25\;keV}}=0.29$, while the absorption ratios $\left(\frac{Q_{40\;keV}}{Q_{25\;keV}}\right)$ were calculated using Equation (2). The dual-energy technique was applied at the image level, in the case of FBP reconstruction, whereas in the case of OSEM was applied directly to the tomographic data (sinograms). Each phantom was simulated twice for each X-ray energy spectrum: microcalcifications were present in the first acquisition, while absent in the second. The breast tissue without microcalcifications was needed to apply Equation (1). Additionally, as usual, blank acquisitions (without the phantom) were performed in air for both energy levels.

Table 4 lists the mean CNR values for phantoms I and II, for both FBP reconstruction and OSEM across all detector configurations under investigation.

**Figure 7:**
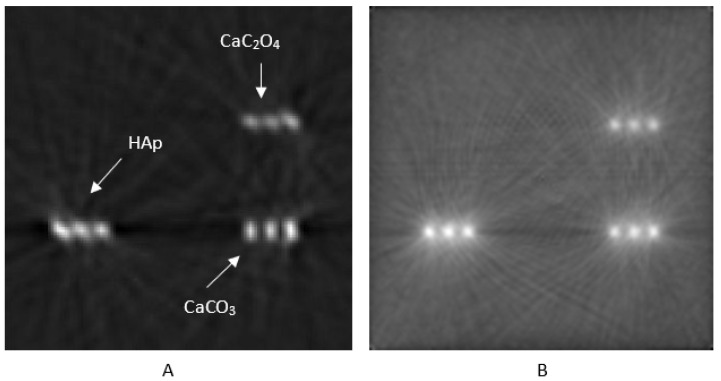
Reconstructed results (slice #50) for breast phantom D (Figure 5), using a LYSO scintillator, reconstructed with (**A**) FBP and (**B**) OSEM, using 24 subsets and 1 iteration.

Figure 7A presents the FBP-reconstructed slice #50 after applying the dual-energy method for phantom IV with types I and II microcalcifications, while Figure 7B shows the corresponding results for OSEM reconstruction, using a LYSO scintillator.

Table 5 lists the mean CNR values for breast phantoms III and IV, for both FBP and OSEM reconstructions, across all detector configurations under investigation.

Table 6 lists the normalized mean squared error for both phantoms III and IV, for both FBP reconstruction and OSEM. NMSREs were calculated based on CsI-reconstructed data as ground truth.

### 3.3. Data Segmentation and Clustering

**Figure 8:**
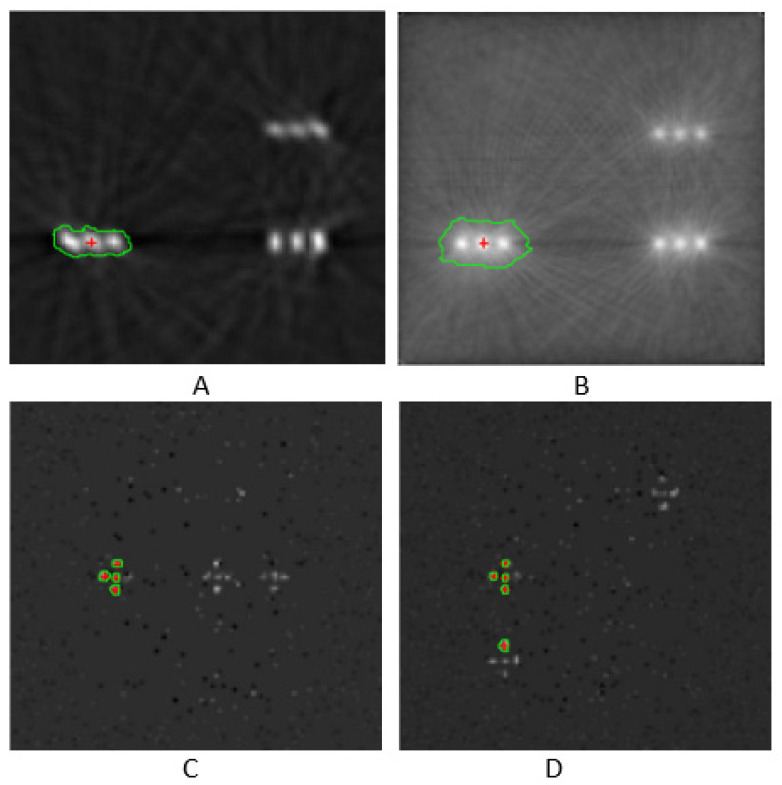
Segmentation results for phantom IV (**A**–**C**) and phantom III (**D**). The setup incorporated a CsI crystal.

**Figure 9:**
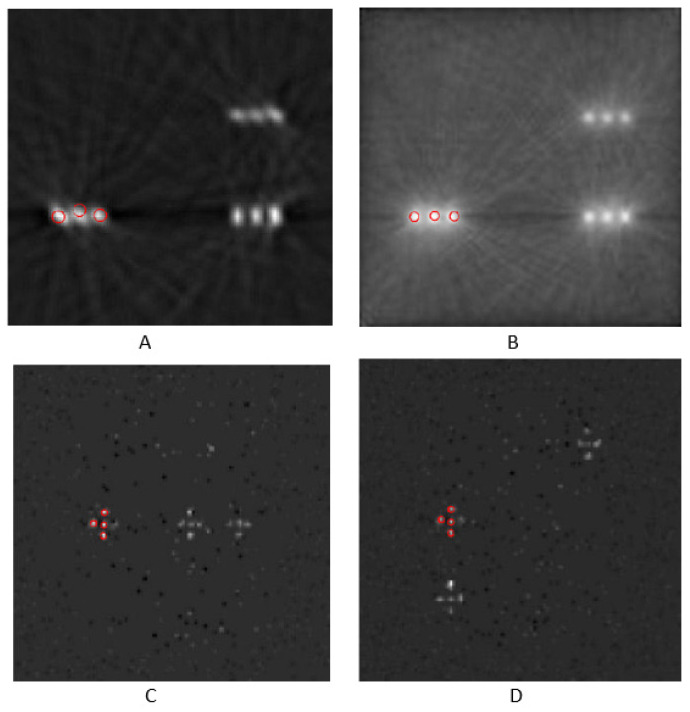
HAp classification with agglomerative clustering of phantom IV (**A**–**C**) and phantom III (**D**). Image (**A**) was reconstructed with FBP, image (**B**) was reconstructed with OSEM, and images (**C**) and (**D**) are planar views. CsI crystal was employed.

The segmentation method used was based on texture evaluation, accompanied by CNR value extraction. Images’ local ROIs were chosen, and standard deviation filtering was applied to detect high-texture regions in the images. Microcalcifications are features that exhibit high-texture characteristics. Then, coherent candidate objects were identified using connected components analysis. The CNR for each candidate object was calculated using the mean value within a 3 × 3 region at its center. Finally, only objects with a CNR greater than 46 were retained for FBP reconstruction, ensuring high detection reliability. The value of CNR > 46 was chosen because HAp’s CNR values, as shown in Table 5, were over 46. Furthermore, the value of CNR > 40 was selected for OSEM-reconstructed images, according to Table 5. The result of this segmentation method for phantom IV, using FBP and OSEM reconstruction, is shown in Figure 8. The specific segmentation algorithm identified HAp microcalcifications for the planar images related to phantoms IV and III, as shown in Figure 8. However, there were also limitations, such as in the example of Figure 8C, where the algorithm misclassified a CaCO_3_ microcalcification as HAp for phantom III due to the high CNR it exhibited in the HAp’s vicinity. To avoid detection errors with this methodology, a preliminary estimation of the CNRs is required so that appropriate thresholds for the contrast-to-noise ratio can be set. The use of a neural network, such as a well-trained U-Net [67,68], might offer greater effectiveness in the characterization of HAp. However, when the suggested segmentation methodology is followed by agglomerative clustering, the mischaracterization of CaCO_3_ as HAp microcalcifications is avoided (Figure 9).

After the segmentation step, agglomerative hierarchical clustering was applied. This clustering method first detected the centroids of desired image features. Then, it clustered objects that were at a predefined maximum specific distance among them. The Euclidean distance was calculated between the feature’s centroids. The maximum object allowable distance was set to 30 pixels. In general, the hyperparameter of maximum allowable distance determines cluster spatial compactness. It also impacts the number of clusters that will be formed. Clustering is based on spatial characteristics, assuming a mono or average object linkage. The latter depends on the internal implementation of the agglomerative spatial clustering function. The criterion of maximum allowable distance makes agglomerative hierarchical clustering independent of a priori knowledge of clusters’ number. Therefore, the aforementioned methodology is suitable for microcalcification clustering, as it recognizes image features with almost identical CNR values that may be spatially placed into clusters, such as HAp microcalcifications [64,65,66,67,68].

The detector configurations used to determine the optimal one included BGO, LSO, LYSO, and LaBr_3_ for scintillators; CZT for semiconductors; and GAGG for ceramic detectors. The subsequent conversion of the detection signal into an electrical pulse, followed by the preprocessing of the electrical pulses, remained the same for all the different detector arrangements under investigation.

### 3.4. Polyenergetic Model

Concerning polyenergetic approaches, studying the application of two energy spectra with maximum energies of 25 keV and 40 keV, respectively, requires one to calculate the number of photons that reach the microcalcifications after passing through an air layer and a few millimeters of water or breast tissue. Additionally, any attenuation of the X-ray beams due to metallic filters—specifically 0.05 mm of rhodium (Rh) and 0.1 mm of aluminum (Al)—at the X-ray tube output must be considered. Consequently, the number of photons reaching the microcalcifications as a function of the photon energy is given by Equation (9):

(9) $$I_{o\;microcalcifications}(E)=I_{o}(E)e^{-(\mu_{filter}(E)x_{filter}+\mu_{air}\left(E\right)x_{air}{+\mu }_{tissue}(E)x_{tissue})}$$

The attenuation coefficients for each material as a function of the photon energy were obtained from the NIST database [52]. The filter thicknesses were 0.05 mm for Rh and 1 mm for Al. The air layer thickness was 141 mm, while the tissue (water or breast) thickness was 5.25 mm for Ca_2_CO_4_ microcalcifications and 10.75 mm for CaCO_3_ and HAp. The ratio $\frac{\mu_{40\;keV}}{\mu_{25\;keV}}$ was calculated for each phantom and each type of microcalcification using Equation (3), revealing that $\frac{\mu_{40\;keV}}{\mu_{25\;keV}}=0.7$ for all types of microcalcifications. Table 7 presents the CNRs for phantom III and breast phantom IV, reconstructed using FBP and OSEM, for all the detector configurations under evaluation. The conclusions did not change for the case of phantoms I and II, so only the results for the phantoms in Figure 4 are presented.

## 4. Discussion

**Figure 10:**
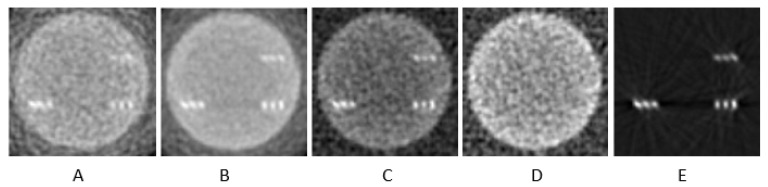
Dual-energy subtraction technique: (**A**) image at low energy, (**B**) image at high energy, (**C**) subtraction of the two images (**A**,**B**,**D**) subtraction of the background, and (**E**) final subtraction between images C and D. CZT detector was used, and images were reconstructed with FBP.

In dual-energy subtraction imaging, tissue structures are canceled out or at least significantly reduced (Figure 10), leading to a much higher CNR value. The advantage of dual-energy imaging lies in the elimination of or substantial reduction in background tissue structures, so they do not overshadow or limit the detection and visualization of microcalcifications. However, the drawback of the dual-energy technique is the reduction in the CNR due to increased noise resulting from the subtraction processing, although CNR values can reach values between 50 and 70 [53]. Moreover, with the introduction of photon-counting detectors in X-ray computed tomography, it has become feasible to acquire the two energy spectra during the same examination needing to switch kV or perform successive scans. This detector technology can be combined with a suitable energy discriminator board, which may contain two or more electronic circuits (each with a separate energy threshold) to separate the X-ray energy spectra. As a result, with the same radiation dose [22], it is possible to obtain both energy spectra and apply dual-energy techniques for further processing of the medical data derived from the two different energy spectra.

In case it is impossible to use the previous examination where microcalcifications are not present, concerning clinical application, usually due to a lack of previous data, the second part of Equation (2) can be avoided, namely, $\{\log\left(\frac{N_{no\;microcalcification,40\;keV}}{N_{o,40\;keV}}\right)-\left(\frac{\mu_{40\;keV}}{\mu_{25\;keV}}\right)\times \left(\frac{Q_{40\;keV}}{Q_{25\;keV}}\right)\log\left(\frac{N_{no\;microcalcification,25\;keV}}{N_{o,25\;keV}}\right)\}$. Instead, a multiplicative empirical factor [9] can be applied in the first part of Equation (1), which can result in Equation (10):

(10) $${de}_{im}=\left\{\log\left(\frac{N_{40\;keV}}{N_{o,40\;keV}}\right)-k\left(\frac{\mu_{40\;keV}}{\mu_{25\;keV}}\right)\times \left(\frac{Q_{40\;keV}}{Q_{25\;keV}}\right)\log\left(\frac{N_{25\;keV}}{N_{o,25\;keV}}\right)\right\}$$

where k stands for the empirical multiplicative factor.

Regarding CNRs, the ceramic scintillator GAGG tends to yield high CNR values, surpassing those of the CsI scintillator, and offers advantages when combined with SiPMs. The LYSO scintillator appears capable of enhancing the reconstructed image using FBP or OSEM, and it is non-hygroscopic. The CZT semiconductor in the monoenergetic beam model that was used provides satisfactorily high contrast-to-noise ratios when used with FBP image reconstruction. In conclusion, replacing the CsI detector in cone-beam X-ray computed tomography systems with scintillators of higher densities and atomic numbers can improve image quality, whether in planar mode or tomographic data acquisition. The ceramic GAGG detector offers benefits in terms of image quality and its ease of fabrication. The CZT semiconductor also appears capable of improving the contrast-to-noise ratio while eliminating the need for an intermediate device to convert light signals into electrical pulses. CZT is already incorporated into clinical systems [69]. Recent scientific research on CZT performance improvement has focused on surface passivation, electrode optimization, readout electronics, compact and scalable designs, and the production of thicker CZT crystals for high-energy applications [70,71]. CZT crystals are preferable for their energy resolution, direct conversion, fast response to external irradiation, and compact detector designs. CZT can be the right choice, especially in high-count-rate applications. Additionally, the lack of CZT application in CBCT is not due to imaging performance but, rather, the difficulty and cost of fabricating large-area, highly uniform CZT detectors [72]. Conversely, GAGG detectors are popular for their high light yield, reasonable timing, and good stopping power [72]; moreover, they are more cost-effective compared with CZT detectors. GAGG is a promising scintillation material, proposed as an alternative to CsI. However, it shows an unusual afterglow under specific exposure conditions [73]. CsI crystal is not expensive, is easy to grow in large volumes, exhibits an adequately high light yield, and is easily integrated into CBCT systems. However, it is characterized by longer decay, lower energy resolution, and hygroscopicity.

The CNR is a widely used, reliable metric of image quality assessment. It shows structure detectability against noise and is the metric of choice in evaluating detector or reconstruction efficiency [74]. CNR values depend on both the size and number of regions of interest (ROIs). The background ROIs were selected near the microcalcifications. Randomization and an increase in the number of background ROIs may further improve the contrast-to-noise ratios. More metrics will be examined in a future work, where ground-truth could be available, such as the structural similarity index (SSIM) [74,75], the correlation coefficient (CC) [76], and the Dice coefficient (Dice) [77], among others, to investigate dual-energy reconstructed images, along with human observers and different simulated phantom geometries, such as d-prime (d′), the area under the curve (AUC), a channelized Hotelling observer (CHO), and so on [78].

As far as NRMSE concerns, the conclusions are similar to CNR findings. GAGG presents the smallest NRMSE for reconstruction data of Phantoms III and IV, while CZT shows an adequately small NRMSE in comparison to CsI data, which were taken as ground truth.

Calcium hydroxyapatite (Ca_10_(PO_4_)_6_(OH)_2_), or HAp, belongs to type II microcalcifications. It contains phosphorus in addition to calcium, and it exhibits high X-ray absorption coefficients for energies in the range of 25–40 keV. Due to its importance in breast imaging, two breast phantoms were simulated, containing HAp, CaCO_3_, and CaC_2_O_4_. Calcium hydroxyapatite (HAp) showed high CNR values for both FBP and OSEM reconstructions across all evaluated detector configurations. HAp has a higher density and greater electron density than the other two types of microcalcifications, resulting in expected CNRs.

In DECT methodology, the energy spectral selection is crucial for image features’ characterization, such as microcalcifications’ clustering. The chosen energy windows determine the desired tissue contrast enhancement. The dual-energy approach is used in mammography and CBCT. In mammography, the typical tube voltages for low- and high-energy spectra are 26–30 kVp and 45–49 kVp, respectively. Commonly found target/filter combinations are Mo/Mo or Mo/Rh for low energy and Rh/Rh or W/Rh for high energy. Tungsten targets are favorable in modern systems because of their high output and spectral flexibility [79]. In CBCT systems, especially for breast or small-animal imaging, broader voltage ranges are used—typically 40–60 kVp for low energy and 80–120 kVp for high energy—often accompanied by additional spectral filtration (e.g., copper or tin filters) to enhance spectral separation [78,79,80,81,82]. According to the literature, monoenergetic beams in the range of 25–30 keV for low energy and 40–45 keV for high energy are preferable in simulation experiments, usually because of the high contrast that HAp exhibits due to its distinct attenuation profile at these energies [37]. For example, if energy beams’ separation is sufficient in GATE-based simulations, then HAp demonstrates high CNR values, and the dual-energy process results in adequate material classification accuracy [83]. More advanced systems employing photon-counting detectors achieve spectral discrimination using multiple energy thresholds (e.g., 20–35 keV and 35–60 keV), eliminating the need for sequential scans or tube voltage switching [83,84]. In all cases, a sufficient energy gap—typically greater than 15 keV—between the low and high spectra is essential for effective material decomposition and optimized CNR performance [85].

Filtration is a key factor in optimizing X-ray spectra for dual-energy imaging in both mammography and breast CBCT. In mammography, low-energy spectra typically employ molybdenum or rhodium filters (e.g., 0.03 mm of Mo or 0.025 mm of Rh [86]) to produce characteristic X-rays at around 17–20 keV, which will enhance soft-tissue contrast [87]. Beam hardening in high-energy windows is achieved by using rhodium or aluminum filters. This will increase the mean photon energy and improve calcification visibility. Moreover, the patient dose is reduced [88]. In breast CBCT, broader energy ranges (40–60 kVp for low energy and 80–120 kVp for high energy) require additional filtration with materials such as copper or tin to effectively separate spectra and enhance material decomposition [84,85]. Proper filtration reduces spectral overlap and unnecessary low-energy photons, which helps improve the contrast-to-noise ratio and decreases radiation exposure [88,89]. Accurate modeling of these filters is also essential in simulation studies to replicate clinical imaging conditions. The presented results are based on the method with which the GATE software simulates the radiation interaction in the filter materials. Although the findings of this work coincide qualitatively with what is expected from theory, other simulation software packages may produce quantitatively different outcomes.

Regarding noise artifacts [89], Figure 7 shows streak artifacts and the beam-hardening effect caused by FBP reconstruction in the vicinity of the microcalcifications’ area. This issue can be mitigated by employing iterative reconstruction algorithms, as demonstrated in Figure 8. Additionally, the relatively large, simulated pixel size of 0.5 mm may contribute to partial volume effect artifacts, particularly in FBP images. Reducing this effect could be achieved by either simulating with a smaller pixel size or using iterative reconstruction methods.

OSEM was implemented using a sequential, non-overlapping approach for selecting subsets. In this method, images are reconstructed from one subset of data and then used as the initial estimate for the reconstruction of the next subset. Completing the reconstruction across all subsets constitutes one full iteration of the algorithm. OSEM serves as an accelerated variant of the EMML (expectation maximization maximum likelihood) algorithm. However, 2 full iterations of OSEM with 24 subsets are not equivalent to 2 full iterations of EMML; the EMML algorithm typically requires around 50 iterations to achieve acceptable results [90]. OSEM was chosen to be implemented with 24 subsets and one iteration, because the schema of 24 subsets and two iterations exhibited similar CNR values. It was better to speed up the reconstruction process.

**Figure 11:**
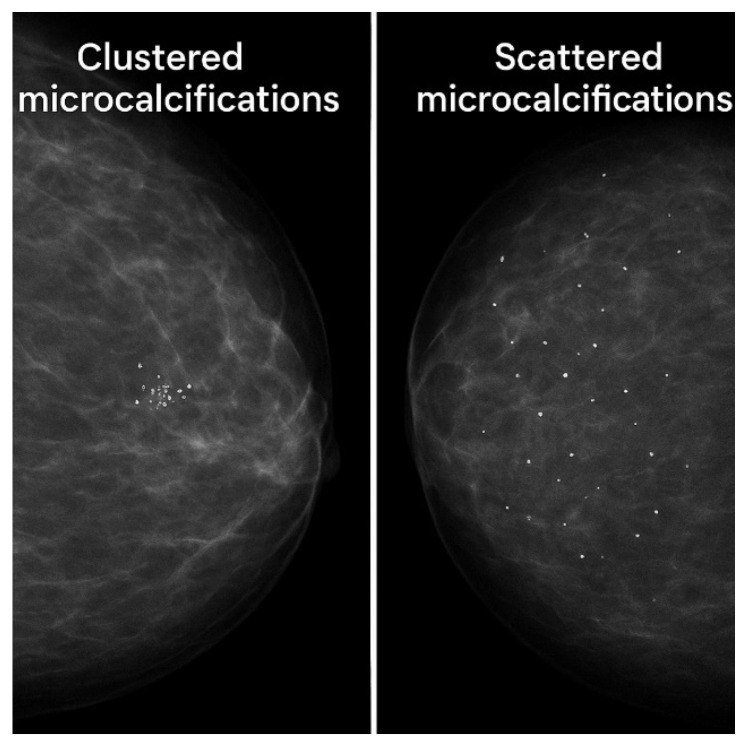
A simulated breast mammographic image with clustered and scattered microcalcifications.

A variety of clustering methods can be used to spatially group image features such as microcalcifications. Agglomerative hierarchical clustering is widely used in medical imaging due to its ease of use, spatial interpretability, and the ability to detect feature clusters based on a distance threshold without requiring a predefined number of groups [91,92,93,94,95,96]. HAp microcalcifications tend to form clusters rather than being scattered all over the breast tissue (Figure 11). DBSCAN, a density-based method, can detect arbitrarily shaped clusters and identify outliers, making it suitable for heterogeneous patterns but sensitive to parameter tuning [96]. Furthermore, K-means clustering assumes spherical objects’ groups and requires prior knowledge of the cluster count, which may not be suitable for dispersed or irregular medical data, though it is computationally efficient [97]. Mean shift and OPTICS offer flexible, nonparametric clustering. Mean shift identifies dense regions without assuming the number of clusters, while OPTICS extends DBSCAN to handle variable densities and offers improved cluster structure detection [98]. Among these, agglomerative clustering is more suitable when spatial proximity and clinical interpretability are priorities in applications such as lesion grouping or microcalcification analysis.

Regarding microcalcification detection and characterization, various quality and quantitative metrics are found in the literature that can assist in diagnostic accuracy and material differentiation. The CNR is a popular and fundamental quality measurement based on contrast differences between microcalcifications and the surrounding background, normalized for background noise [99,100,101]. Spectral discrimination of calcium deposits can also be assessed using energy-dependent contrast difference variables, such as C_l_ and C_h_, which represent the logarithmic ratios of X-ray intensities between breast tissue with and without microcalcifications at low and high energies. These ratios provide valuable information on tissue attenuation characteristics [82,83]. Compositional variances between energies can also be characterized via the dual-energy index (DEI), which can be derived from C_l_ and C_h_. Differentiation between microcalcifications and efficient elemental decomposition can also be determined by estimating the calcium-to-phosphorus mass ratio. HAp contains phosphorus and theoretically exhibits a calcium-to-phosphorus mass ratio of $m_{Ca}/m_{P}$, different from the other microcalcifications. Conversely, calcium hydronated or calcium carbonate completely lacks phosphorus. These compositional differences are particularly valuable for assessing the malignant potential of detected microcalcifications.

Compared with the monoenergetic beam model, the overall behavior of the contrast-to-noise ratios for the three types of microcalcifications and for both the water and breast tissue phantoms, respectively, does not significantly differ.

The proportions of the breast phantom do not represent the full volume of a human breast. Instead, they correspond to a small region that mimics breast tissue. The phantom is specifically designed to evaluate the effectiveness of different detector materials in distinguishing structures with dimensions comparable to the spatial resolution of the micro-CBCT system, within a confined area of simulated breast tissue. The breast phantoms have a higher density than usual breast tissue; therefore, they simulate dense breast tissue.

## 5. Conclusions

In this work, a dual-energy technique was applied to image data obtained from the simulation of the irradiation of a breast tissue phantom with various micro-CBCT system configurations. Seven different detector configurations were simulated and evaluated in terms of the image quality, as derived from local CNRs. The dual-energy methodology was applied to breast phantom data containing microcalcifications of types I and II. CNR values were used, accompanied by texture and entropy segmentation techniques combined with agglomerative clustering. Among the seven detector configurations, CZT and GAGG crystals were found to be the best alternatives to a standard CsI scintillator. They presented high CNRs for all types of microcalcifications, higher than CsI’s CNR values, regardless of whether a monoenergetic or polyenergetic model was adopted.

## Figures and Tables

**Table 2:** Mean CNR values for the detector configurations of this study for oblique-view acquisition for phantoms I and II using the dual-energy method.

Detector Material	CNR_mean_			
	Ca_2_CO_4_		CaCO_3_	
	Phantom I	Phantom II	Phantom I	Phantom II
CsI	3.03	5.08	32.03	29.76
BGO	3.77	4.44	45.74	24.29
LSO	2.99	4.90	13.09	29.08
LYSO	2.76	4.75	40.15	32.16
LaBr_3_	3.12	4.67	50.55	29.62
CZT	2.89	4.57	30.25	25.53
GAGG	2.96	4.92	39.83	25.77

**Table 3:** Mean CNRs for phantoms III and IV in planar mode.

Detector Material	CNR_mean_					
	CaC_2_O_4_		CaCO_3_		Hap	
	Phantom III	Phantom IV	Phantom III	Phantom IV	Phantom III	Phantom IV
CsI	9.82	9.69	19.58	21.14	24.55	32.40
BGO	10.09	5.98	18.37	11.57	25.64	31.20
LSO	10.85	6.22	17.13	12.84	28.43	31.51
LYSO	10.78	5.90	18.01	12.10	20.26	27.40
LaBr_3_	9.82	8.11	14.73	9.86	18.98	25.15
CZT	14.08	10.57	22.41	26.89	35.10	42.63
GAGG	16.51	11.27	33.37	27.87	37.14	44.09

**Table 4:** Mean CNR values for phantom I and breast tissue phantom II, for both FBP reconstruction and OSEM, across all detector configurations under study.

Detector Material	CNR_mean_							
	Ca_2_CO_4_				CaCO_3_			
	Phantom I		Phantom II		Phantom I		Phantom II	
	FBP	OSEM	FBP	OSEM	FBP	OSEM	FBP	OSEM
CsI	12.63	24.83	6.90	29.55	24.93	31.34	14.00	37.18
BGO	13.69	20.72	7.35	29.97	28.15	37.87	13.44	26.24
LSO	13.21	25.59	7.56	28.87	25.45	28.84	14.96	30.00
LYSO	13.40	21.33	7.37	28.44	26.72	33.79	15.03	31.01
LaBr_3_	12.85	22.82	6.88	27.06	27.94	31.65	13.56	30.12
CZT	12.85	28.15	7.02	29.29	24.68	33.62	16.01	25.51
GAGG	12.22	20.71	7.51	30.41	26.74	33.93	14.85	38.50

**Table 5:** Mean CNR values for breast phantoms III and IV (Figure 5) for both FBP reconstruction and OSEM, across all detector configurations under examination.

CNR_mean_						
Detector Material	FBP					
	CaC_2_O_4_		Hap		CaCO_3_	
	Phantom III	Phantom IV	Phantom III	Phantom IV	Phantom III	Phantom IV
CsI	16.97	13.58	70.41	54.91	44.59	39.65
BGO	15.16	13.04	61.04	61.13	40.22	37.60
LSO	16.73	11.91	72.15	54.50	46.29	36.45
LYSO	18.76	12.66	63.40	57.78	40.20	36.75
LaBr_3_	16.85	13.71	76.84	52.95	39.78	36.58
CZT	17.76	14.11	75.92	58.07	44.03	43.16
GAGG	22.29	20.19	85.39	57.97	47.14	44.68
**Detector Material**	**OSEM**					
	**CaC_2_O_4_**		**Hap**		**CaCO_3_**	
	**Phantom III**	**Phantom IV**	**Phantom III**	**Phantom IV**	**Phantom III**	**Phantom IV**
CsI	15.10	15.71	56.30	49.02	26.34	22.24
BGO	15.03	13.49	57.52	50.73	25.61	24.30
LSO	19.17	13.94	55.42	47.83	26.46	21.98
LYSO	17.02	13.73	55.33	43.32	28.63	23.65
LaBr_3_	12.89	13.28	46.28	44.47	20.85	21.81
CZT	17.45	17.60	60.28	51.82	32.30	23.37
GAGG	18.62	16.30	47.28	53.37	29.40	23.79

**Table 6:** NMSRE values for breast phantoms III and IV (Figure 5) for both FBP reconstruction and OSEM, across all detector configurations under examination.

NMSRE				
		FBP		
Detector Material	Phantom III #slice 25	Phantom III #slice 50	Phantom III #slice 70	Phantom IV #slice 50
BGO	0.0110	0.0084	0.0072	0.0102
LSO	0.0147	0.0050	0.0061	0.0064
LYSO	0.0120	0.0126	0.0073	0.0098
LaBR_3_	0.0099	0.0158	0.0080	0.0146
CZT	0.0072	0.0068	0.0083	0.0106
GAGG	0.0103	0.0145	0.0062	0.0066
		**OSEM**		
**Detector Material**	**Phantom III** **#slice 25**	**Phantom III** **#slice 50**	**Phantom III** **#slice 70**	**Phantom IV** **#slice 50**
BGO	0.0115	0.0105	0.0101	0.0091
LSO	0.0144	0.0114	0.0162	0.0066
LYSO	0.0132	0.0114	0.0178	0.0093
LaBR_3_	0.0150	0.0104	0.0149	0.0096
CZT	0.0135	0.0100	0.0171	0.0095
GAGG	0	0	0	0

**Table 7:** Mean CNR values for breast phantoms III and IV (Figure 5), for both FBP reconstruction and OSEM across all detector configurations under study, using the polyenergetic model for the two energy spectra of maximum energies of 25 keV and 40 keV, respectively.

CNR_mean_						
Detector Material	FBP					
	CaC_2_O_4_		Hap		CaCO_3_	
	Phantom III	Phantom IV	Phantom III	Phantom IV	Phantom III	Phantom IV
CsI	15.58	12.70	60.49	47.55	43.69	37.00
BGO	13.43	12.35	50.91	51.73	38.49	34.68
LSO	14.90	11.13	62.95	47.99	45.72	33.04
LYSO	16.88	11.91	53.3	50.12	38.42	33.90
LaBr_3_	15.61	12.74	66.41	46.77	37.60	33.69
CZT	16.14	13.63	65.00	50.02	42.59	39.91
GAGG	20.02	11.53	69.94	50.62	45.66	34.19
**Detector Material**	**OSEM**					
	**CaC_2_O_4_**		**Hap**		**CaCO_3_**	
	**Phantom III**	**Phantom IV**	**Phantom III**	**Phantom IV**	**Phantom III**	**Phantom IV**
CsI	14.62	14.97	38.87	39.54	26.60	26.45
BGO	14.33	13.03	51.24	44.13	25.12	22.93
LSO	19.42	13.36	32.61	41.22	25.10	23.32
LYSO	17.49	13.06	48.83	37.44	26.76	22.64
LaBr_3_	12.57	12.76	38.83	40.53	21.52	21.23
CZT	16.80	12.43	49.68	45.33	28.62	23.21
GAGG	17.98	12.95	39.11	42.89	29.18	24.76

## Data Availability

The data are contained within this article.

## Abbreviations

The following abbreviations were used in this manuscript:

CBCT	Cone-beam computed tomography
CNR	Contrast-to-noise ratio
SNR	Signal-to-noise ratio
FBP	Filtered backprojection algorithm
OSEM	Ordered subsets expectation maximization algorithm
TDLU	Terminal ductal lobular units
BI-RADS	Breast Imaging Reporting and Database System
DQE	Detective quantum efficiency
DECT	Dual-energy computed tomography
PCD	Photon-counting detector
CT	Computed tomography
NIST	National Institute of Standards and Technology
GATE	GEANT4 application for tomographic emission
GEANT4	Geometry and tracking 4
LOR	Line of response
EMML	Expectation maximization maximum likelihood
DBSCAN	Density-based spatial clustering of applications with noise
OPTICS	Ordering points to identify the clustering structure
DEI	Dual-energy index

## References

[1] European Commission (2023). Breast Cancer in the EU.

[2] Yurdusev A.A., Adem K., Hekim M. (2023). Detection and classifications in mammograms images using difference filter and Yolov4 deep learning model. Biomed. Signal Process. Control.

[3] Birk U., Diekmann F. (2010). Digital Mammography.

[4] Eisenbrey J.R., Dave J.K., Forsberg F. (2016). Recent technological advancements in breast ultrasound. Ultrasonics.

[5] Baker J.A., Lo J.Y. (2011). Breast tomosynthesis. Acad. Radiol..

[6] Gilbert F.J., Pinker-Domenig K., Hodler J., Kubik-Huch R.A., von Schulthess G.K. (2019). Diagnosis and Staging of Breast Cancer: When and How to Use Mammography, Tomosynthesis, Ultrasound, Contrast-Enhanced Mammography, and Magnetic Resonance Imaging. Diseases of the Chest, Breast, Heart and Vessels 2019–2022: Diagnostic and Interventional Imaging.

[7] O’Grady S., Morgan M. (2018). Microcalcifications in breast cancer: From pathophysiology to diagnosis and prognosis. Biochim. Biophys. Acta Rev. Cancer.

[8] Logullo A.F., Prigenzi K.C.K., Nimir C.C.B.A., Franco A.F.V., Campos M.S.D.A. (2022). Breast microcalcifications: Past, present and future (Review). Mol. Clin. Oncol..

[9] Wilkinson L., Thomas V., Sharma N. (2017). Microcalcification on mammography: Approaches to interpretation and biopsy. Br. J. Radiol..

[10] Bistoni G., Farhadi J., Farhadieh R.D., Bulstrode N.W., Cugno S. (2015). Anatomy and physiology of the breast. Plastic and Reconstructive Surgery. Approaches and Techniques.

[11] Martini N., Koukou V., Fountos G., Michail C., Bakas A., Kandarakis I., Speller R., Nikiforidis G. (2017). Characterization of breast calcification types using dual energy X-ray method. Phys. Med. Biol..

[12] Martini N., Koukou V., Michail C., Fountos G. (2021). Mineral Characterization in Human Body: A Dual Energy Approach. Crystals.

[13] Arancibia Hernández P.L., Taub Estrada T., López Pizarro A., Díaz Cisternas M.L., Sáez Tapia C. (2016). Calcificaciones mamarias: Descripción y clasificación según la 5.a edición BI-RADS. Rev. Chil. Radiol..

[14] O’Connell A.M., Marini T.J., Kawakyu-O’Connor D.T. (2021). Cone-Beam Breast Computed Tomography: Time for a New Paradigm in Breast Imaging. J. Clin. Med..

[15] Dagnall K.A., Conley A.M., Yoon L.U., Rajeev H.S., Lee S.-H., Choi J.J. (2022). Ytterbium-doped cesium lead chloride perovskite as an X-ray scintillator with high light yield. ACS Omega.

[16] Mikhailik V.B., Kapustyanyk V., Tsybulskyi V., Rudyk V., Kraus H. (2015). Luminescence and scintillation properties of CsI: A potential cryogenic scintillator. Phys. Status Solidi B.

[17] Zaidi H. (2014). Molecular Imaging of Small Animals: Instrumentation and Applications.

[18] García-Jiménez G., Cabanelas P., González-Caamaño D., Alvarez-Pol H., Vicente-Pardal M.A., Benlliure J., Cederkäll J., Cortina-Gil D., Feijoo-Fontán M., Graña-González A. (2024). Study of scintillation properties and performance of CsI(Tl) detectors over time. Nucl. Instrum. Methods Phys. Res. A.

[19] Tian C., Liu S., Xie Y., Guo L., Chen D., Liu Y., Zhong Z. (2019). Study on the mechanism of afterglow in CsI:Tl and the afterglow suppression in CsI: Tl, Eu. J. Radioanal. Nucl. Chem..

[20] Michail C., Liaparinos P., Kalyvas N., Kandarakis I., Fountos G., Valais I. (2024). Phosphors and scintillators in biomedical imaging. Crystals.

[21] Danielsson M., Persson M., Sjölin M. (2021). Photon-counting X-ray detectors for CT. Phys. Med. Biol..

[22] Noel A., Thibault F. (2004). Digital detectors for mammography: The technical challenges. Eur. Radiol..

[23] Shi L., Bennett N.R., Shapiro E., Colbeth R.E., Star-Lack J., Lu M., Wang A.S. (2020). Comparative study of dual energy cone-beam CT using a dual-layer detector and kVp switching for material decomposition. Medical Imaging 2020: Physics of Medical Imaging.

[24] Johnson T.R.C. (2012). Dual-Energy CT: General Principles. AJR.

[25] Goo H.W., Goo J.M. (2017). Dual-energy CT: New Horizon in Medical Imaging. Korean J. Radiol..

[26] Solomon J., Mileto A., Ramirez-Giraldo J.C., Samei E. (2017). Diagnostic performance of an advanced modeled iterative reconstruction algorithm for low-contrast detectability with a third-generation dual-source multidetector CT scanner: Potential for radiation dose reduction in a multireader study. Radiology.

[27] Ay M.R., Zadeh M.A., Ghadiri H., Zaidi H. (2022). Comparative assessment of image quality in TrueFidelity deep-learning image reconstruction and adaptive statistical iterative reconstruction-V in abdominal CT imaging. Eur. Radiol. Exp..

[28] Ghammraoui B., Badal A. (2017). Evaluation of potential benefit of spectroscopic breast CT using a photon counting detector for imaging of Iodine and Calcium contrast agents. Phys. Med. Biol..

[29] Andersson I., Ikeda D.M., Zackrisson S. (2008). Breast tomosynthesis: State-of-the-art and review of the literature. Breast Cancer Res. Treat..

[30] Tagliafico A., Astengo D., Mariscotti G., Durando M., Rousset M., Rubini G., Calabrese M., Valdora F., Houssami N. (2015). Mammographic density: Comparison of visual assessment with fully automated estimation. Radiol. Med..

[31] Brodersen J., Siersma V.D. (2013). Long-term psychosocial consequences of false-positive screening mammography. Ann. Fam. Med..

[32] Martini N., Fountos G., Michail C., Bakas A., Nikiforidis G., Speller R. (2015). Experimental investigation and Monte Carlo simulation of a dual energy mammographic system for calcification characterization. Nucl. Instrum. Methods Phys. Res. A.

[33] Fountos G., Michail C., Kalyvas N., Koukou V., Bakas A., Nikiforidis G. (2018). Evaluation of breast material decomposition using synthetic dual energy images. Biocybern. Biomed. Eng..

[34] Ghammraoui B., Badal A., Kanarek D., Myers K. (2018). Dual-energy imaging for improved detection and classification of microcalcifications in digital breast tomosynthesis. Med. Phys..

[35] Vázquez A., Arce P., Rato J.M., Díez S., Fernández M., Zanca F., Sánchez-González J. (2020). Simulation study of dual-energy contrast-enhanced breast CT for calcification discrimination. Med. Phys..

[36] Martinez N., Koukou V., Bakas A., Fountos G., Speller R., Nikiforidis G. (2020). A novel dual-energy mammographic algorithm to decompose and classify microcalcifications based on their effective atomic number. Phys. Med..

[37] Sechopoulos I. (2013). A review of breast tomosynthesis. Part I. The image acquisition process. Med. Phys..

[38] Pepin C.M., Berard P., Perrot A.-L., Pepin C., Houde D., Lecomte R., Melcher C.L., Dautet H. (2004). Properties of LYSO and Recent LSO Scintillators for Phoswich PET Detectors. IEEE Trans. Nucl. Sci..

[39] Van der Sar S., Brunner S., Schaart D. X-ray Photon-Counting Using Silicon Photomultiplier-Based Scintillation Detectors at High X-ray Tube Currents. Proceedings of the SPIE 12031, Medical Imaging 2022: Physics of Medical Imaging.

[40] Berg E., Cherry S.R. (2018). Innovations in Instrumentations for positron emission tomography. Semin Nucl. Med..

[41] Zhu Y., Qian S., Wang Z., Guo H., Ma L., Wang Z., Wu Q. (2020). Scintillation properties of GAGG: Ce ceramic and single crystal. Opt. Mater..

[42] Dey Chaudhuri S., Banerjee D., Bhattacharjee T., Wasim Raja S., Acharya R., Pujari P.K. (2020). Performance Study of LaBr_3_:Ce Detectors Coupled to R2083 PM Tube for Energy and Timing Characteristics. J. Radioanal. Nucl. Chem..

[43] Tseremoglou S., Michail C., Valais I., Ninos K., Bakas A., Kandarakis I., Fountos G., Kalyvas N. (2024). Optical Photon Propagation Characteristics and Thickness Optimization of LaCl_3_:Ce and LaBr_3_:Ce Crystal Scintillators for Nuclear Medicine Imaging. Crystals.

[44] Opanasyuk A., Kurbatov D., Znamenshchykov Y., Diachenko O., Ivashchenko M., Korotcenkov G. (2023). CdTe-/CdZnTe-Based Radiation Detectors. Handbook of II-VI Semiconductor-Based Sensors and Radiation Detectors.

[45] Herman G.T. (1980). Image Reconstruction from Projections: The Fundamentals of Computed Tomography.

[46] Yanagida T. (2018). Inorganic scintillating materials and scintillation detectors. Proc. Jpn. Acad. Ser. B.

[47] Xie S., Zhang X., Zhang Y., Ying G., Huang Q., Xu J., Peng Q. (2020). Evaluation of Various Scintillator Materials in Radiation Detector Design for Positron Emission Tomography (PET). Crystals.

[48] OpenGATE Collaboration GATE Documentation—GAM Documentation. https://opengate.readthedocs.io/_/downloads/en/v9.0/pdf/.

[49] Fountos G., Michail C., Koukou V., Bakas A., Nikiforidis G. (2018). A simulation study for dual-energy mammographic imaging. Radiat. Phys. Chem..

[50] Koukou V., Martini N., Michail C., Sotiropoulou P., Kalyvas N., Kandarakis I., Nikiforidis G., Fountos G. (2015). Optimum filter selection for Dual Energy X-ray Applications through Analytical Modeling. J. Phys. Conf. Ser..

[51] Bliznakova K., Kolitsi Z., Pallikarakis N. (2006). Dual-energy mammography: Simulation studies. Phys. Med. Biol..

[52] National Institute of Standards and Technology (NIST) XCOM: Element/Compound/Mixture. https://physics.nist.gov/PhysRefData/Xcom/html/xcom1.html.

[53] Lemacks M.R., Kappadath S.C., Shaw C.C., Liu X., Whitman G.J. (2002). A dual-energy subtraction technique for microcalcification imaging in digital mammography—A signal-to-noise analysis. Med. Phys..

[54] Feldkamp L.A., Davis L.C., Kress J.W. (1984). Practical Cone-Beam Algorithm. J. Opt. Soc. Am. A.

[55] Pan X., Xia D., Zou Y., Yu L. (2004). A unified analysis of FBP-based algorithms in helical cone-beam and circular cone- and fan-beam scans. Phys. Med. Biol..

[56] Hudson H.M., Larkin R.S. (1994). Accelerated Image Reconstruction Using Ordered Subsets of Projection Data. IEEE Trans. Med. Imaging.

[57] Trevisan A.C., Raed M.D., Tumas V., Alexandre-Santos L., Pitella F.A., Itikawa E.N., Silvah J.H., Kato M., Martinez E.Z., Achcar J.A. (2020). Comparison between OSEM and FBP reconstruction algorithms for the qualitative and quantitative interpretation of brain DAT-SPECT using an anthropomorphic striatal phantom: Implications for the practice. Res. Biomed. Eng..

[58] Cherry S.R., Sorenson J.A., Phelps M.E. (2012). Physics in Nuclear Medicine.

[59] Karali E., Michail C., Fountos G., Kalyvas N., Valais I. (2024). Novel Detector Configurations in Cone-Beam CT Systems: A Simulation Study. Crystals.

[60] Moulden B., Kingdom F., Gatley L.F. (1990). The standard deviation of luminance as a metric for contrast in random-dot images. Perception.

[61] Gavrielides M.A., Lo J.Y., Vargas-Voracek R., Floyd C.E. (2000). Segmentation of suspicious clustered microcalcifications in mammograms. Med. Phys..

[62] Zhang X., Homma N., Goto S., Kawasumi Y., Ishibashi T., Abe M., Sugita N., Yoshizawa M. (2013). A hybrid image filtering method for computer-aided detection of microcalcification clusters in mammograms. J. Med. Eng..

[63] Kim H., Lee M., Kim D., Lee D., Kim H.-J. (2019). Evaluation of photon-counting spectral mammography for classification of breast microcalcifications. Radiat. Phys. Chem..

[64] Ciecholewski M., Kasprzak A., Pociask P. (2019). A Novel Method for Detecting Clusters of Microcalcifications in Digital Mammograms Based on Multi-Stage Feature Enhancement. Algorithms.

[65] Dada E.G., Oyewola D.O., Misra S. (2024). Computer-aided diagnosis of breast cancer from mammogram images using deep learning algorithms. J. Electr. Syst. Inf. Technol..

[66] Gong Y., Giger M.L., Zeng R., Jiang Y. (2011). Computerized Detection of Microcalcification Clusters for Digital Mammography: Image Enhancement Technique Using Contrast-to-Noise Ratio. Med. Phys..

[67] Zhou S., Lin K., Wang Y., Jafari M.H., Qian W. (2014). Clustered Microcalcification Detection Using Graph Theory. BMC Med. Imaging.

[68] Azad R., Aghdam E.K., Zafar M.R., Momeni F., Tajbakhsh N., Zhou S.K., Merhof D., Albarqouni S. (2024). Medical Image Segmentation Review: The Success of U-Net. IEEE Trans. Pattern Anal. Mach. Intell..

[69] Greffier J., Viry A., Robert A., Khorsi M., Si-Mohamed S. (2025). Photon-counting CT systems: A technical review of current clinical possibilities. Diagn. Interv. Imaging.

[70] Mutua J., Di J., Liu L., Zheng X. (2025). CZT-powered photon-counting CT: A revolution in medical imaging. Biomed. Phys. Eng. Express.

[71] Chen H., Li H., Sundaram A.G., Reed M.D., Eger J., Montémont G., Verger L., He Z., Hugg J.W., Abbaszadeh S., Fiederle M., Burger A., James R.B., Payne S.A. (2018). Development of large-volume high-performance monolithic CZT radiation detector. Hard X-Ray, Gamma-Ray, and Neutron Detector Physics XX.

[72] Maldera A., De Marco P., Colombo P.E., Origgi D., Torresin A. (2017). Digital breast tomosynthesis: Dose and image quality assessment. Phys. Medica.

[73] Dimitrakopoulos A., Michail C., Valais I., Fountos G., Kandarakis I., Kalyvas N. (2025). Experimental Evaluation of GAGG: Ce Crystalline Scintillator Properties Under X-Ray Radiation. Crystals.

[74] Nocetti D., Villalobos K., Wunderle K. (2023). Physical image quality metrics for the characterization of x-ray systems used in fluoroscopy-guided pediatric cardiac interventional procedures: A systematic review. Children.

[75] Wang Z., Bovik A.C., Sheikh H.R., Simoncelli E.P. (2004). Image quality assessment: From error visibility to structural similarity. IEEE Trans. Image Process..

[76] Press W.H., Flannery B.P., Teukolsky S.A., Vetterling W.T. (1992). Numerical Recipes in C: The Art of Scientific Computing.

[77] Dice L.R. (1945). Measures of the amount of ecologic association between species. Ecology.

[78] Fan M., Thayib T., McCollough C., Yu L. (2023). Accurate and efficient measurement of channelized Hotelling observer-based low-contrast detectability on the ACR CT accreditation phantom. Med. Phys..

[79] Shrestha S., Vedantham S., Karellas A. (2017). Towards standardization of x-ray beam filters in digital mammography and digital breast tomosynthesis: Monte Carlo simulations and analytical modelling. Phys. Med. Biol..

[80] Li W., Zhang Q., Black D., Ding H., Iribarren C., Shojazadeh A., Molloi S. (2025). Quantification of breast arterial calcification in mammograms using a unet-based deep learning for detecting cardiovascular disease. Acad. Radiol..

[81] Dance D.R., Cristofides S., Maidment A.D.A., McLean I.D., NG K.-H. (2014). Diagnostic Radiology Physics: A Handbook for Teachers and Students.

[82] Carton A.-K., Ullberg C., Maidment A.D.A. (2010). Optimization of a dual-energy contrast-enhanced technique for a photon-counting digital breast tomosynthesis system: II. An experimental validation. Med. Phys..

[83] Kim H., Kim J., Kim K., Kim T. (2020). Discrimination of Benign and Malignant Breast Microcalcifications Using Spectral Mammography: A Monte Carlo Simulation Study with GATE. J. Instrum..

[84] Ghammraoui B., Glick S.J. Classification of breast microcalcifications using spectral mammography. Proceedings of the SPIE Medical Imaging 2017: Physics of Medical Imaging.

[85] Boone J.M., Kwan A.L.C., Seibert J.A., Shah N., Lindfors K.K., Nelson T.R. (2005). Technique Factors and Their Relationship to Radiation Dose in Pendant Geometry Breast CT. Med. Phys..

[86] Symons R., Krauss B., Sahbaee P., Cork T.E., Lakshmanan M.N., Bluemke D.A., Pourmorteza A. (2017). Photon-counting CT for simultaneous imaging of multiple contrast agents in the abdomen: An in vivo study. Med. Phys..

[87] Si-Mohamed S.A., Boccalini S., Lacombe H., Miailhes J., Rodesch P.-A., Leitman V., Cottin V., Boussel L., Douek P. (2021). Spectral Photon-Counting CT Technology in Chest Imaging. J. Clin. Med..

[88] Marsh J.F., Jorgensen S.M., Rundle D.S., Vercnocke A.J., Leng S., Butler P.H., McCollough C.H., Ritman E.L. (2018). Evaluation of a Photon Counting Medipix3RX Cadmium Zinc Telluride Spectral X-ray Detector. J. Med. Imaging.

[89] Schulze R., Heil U., Groβ D., Bruellmann D., Dranischnikow E., Schwanecke U., Schoemer E. (2011). Artefacts in CBCT: A Review. Dentomaxillofac. Radiol..

[90] Shepp L.A., Vardi Y. (1982). Maximum Likelihood Reconstruction for Emission Tomography. IEEE Trans. Med. Imaging.

[91] Ferro S., Bottigliengo D., Gregori D., Fabricio A.S.C., Gion M., Baldi I. (2021). Phenomapping of Patients with Primary Breast Cancer Using Machine Learning-Based Unsupervised Cluster Analysis. J. Pers. Med..

[92] Haka A.S., Shafer-Peltier K.E., Fitzmaurice M., Crowe J., Dasari R.R., Feld M.S. (2005). Diagnosing breast cancer by using Raman spectroscopy. Proc. Natl. Acad. Sci. USA.

[93] El-Naqa I., Yang Y., Wernick M.N., Galatsanos N.P., Nishikawa R.M. (2002). A support vector machine approach for detection of microcalcifications. IEEE Trans. Med. Imaging.

[94] Sahiner B., Petrick N., Chan H.P., Hadjiiski L.M., Paramagul C., Helvie M.A., Gurcan M.N. (2001). Computer-aided characterization of mammographic masses: Accuracy of mass segmentation and its effects on characterization. IEEE Trans. Med. Imaging.

[95] Ester M., Kriegel H.-P., Sander J., Xu X. (1996). A density-based algorithm for discovering clusters in large spatial databases with noise. Proceedings of the 2nd International Conference on Knowledge Discovery and Data Mining (KDD’96), Portland, Oregon, 2–4 August 1996.

[96] MacQueen J. (1967). Some methods for classification and analysis of multivariate observations. Proceedings of the 5th Berkeley Symposium on Mathematical Statistics and Probability, Berkeley, CA, USA, 21 June –18 July 1965 and 27 December 1965–7 January 1966.

[97] Comaniciu D., Meer P. (2002). Mean shift: A robust approach toward feature space analysis. IEEE Trans. Pattern Anal. Mach. Intell..

[98] Ankerst M., Breunig M.M., Kriegel H.P., Sander J. (1999). OPTICS: Ordering points to identify the clustering structure. ACM SIGMOD Rec..

[99] Samei E., Flynn M.J. (2003). An experimental comparison of detector performance for direct and indirect digital radiography systems. Med. Phys..

[100] Kalender W.A. (2006). X-ray computed tomography. Phys. Med. Biol..

[101] Sotiropoulou P.I., Fountos G.P., Martini N.D., Koukou V.N., Michail C.M., Valais I.G., Kandarakis I.S., Nikiforidis G.C. (2015). X-ray dual energy spectral parameter optimization for bone Calcium/Phosphorus mass ratio estimation. J. Phys. Conf. Ser..

